# Viral etiology of community-acquired pneumonia among adolescents and adults with mild or moderate severity and its relation to age and severity

**DOI:** 10.1186/s12879-015-0808-0

**Published:** 2015-02-22

**Authors:** Jiu-Xin Qu, Li Gu, Zeng-Hui Pu, Xiao-Min Yu, Ying-Mei Liu, Ran Li, Yi-Min Wang, Bin Cao, Chen Wang, For Beijing Network for Adult Community-Acquired Pneumonia (BNACAP)

**Affiliations:** Department of Infectious diseases and Clinical Microbiology, Beijing Chao-Yang Hospital, Capital Medical University, Beijing Institute of Respiratory Medicine, No 8 Gongti Nanlu, ChaoYang District, Beijing, 100020 China; Department of Infectious diseases, YanTai Yu Huang-Ding Hospital, Yantai, China; Department of Respiratory Medicine, Capital Medical University; Beijing Institute of Respiratory Medicine; Beijing Key Laboratory of Respiratory and Pulmonary Circulation Disorders, Beijing, China

**Keywords:** Community-acquired pneumonia, Respiratory viral infection, Pneumonia severity index, Adolescent, Adult, Influenza virus A, Adenovirus, Human rhinovirus

## Abstract

**Background:**

Better knowledge of distribution of respiratory viruses (RVs) in adolescents and adults with community-acquired pneumonia (CAP) is needed.

**Methods:**

To investigate the RVs etiology among adolescents and adults with CAP, according to age and pneumonia severity index (PSI), a multi-center, prospective study was conducted from November 2010 to April 2012. Fifteen RVs were tested by polymerase chain reaction (PCR). Bacteria were detected by urinary antigen, conventional culture and PCR.

**Results:**

Mean (SD) age and median (IQR) PSI score of 954 patients enrolled was 45.2 (19.5) years (range 14–94) and 42 (36). RVs were found in 262 patients (27.5%): influenza virus A (IFV A, 9.9%) comprised of pandemic H1N1 (6.7%) and seasonal H3N2 (3.5%), human rhinovirus (4.3%), adenovirus (4.2%), human metapneumovirus (1.8%), parainfluenza virus 1, 3 and 2 (1.7%, 1.5% and 1.2%). Influenza virus B, enterovirus, respiratory syncytial virus, human coronavirus and parainfluenza virus 4 were rarely detected (<1%). Frequency of IFV A was highest among patients aged between 45–64 years (p < 0.001), while adenovirus among patients aged 14–17 years (p < 0.001), no differences was found in other RVs. The proportion of pandemic H1N1 increased with severity of pneumonia evaluated by PSI (P < 0.05).

**Conclusions:**

The proportion of RVs in CAP is higher than previously reported. IFV A pneumonia are usually found in patients older than 45 years, while, adenovirus pneumonia are common in adolescents and young adults. Pandemic H1N1 virus is still recognized by PSI as a high-severity pathogen. The findings contribute baseline data on viral CAP study in China.

## Background

Community-acquired pneumonia (CAP) remains a common disease associated with significant morbidity and mortality. Mortality varied from <1% to 48% and is associated with advanced age, co-morbid conditions, and CAP severity [1]. Clear etiology is essential for the management of CAP patients [2]. Although CAP guidelines acknowledge respiratory viruses (RVs) as a “cause” of adults pneumonia [3], few recommendations are made regarding management, largely due to the short of data regarding prevalence and clinical severity, as most relevant data concern infants and children [4,5]. In addition, the emergence of severe acute respiratory syndrome (SARS), avian influenza A (H5N1) virus, and the pandemic influenza A 2009 (pH1N1) virus has emphasized the important role of RVs as causes of CAP [6]. Thus, much better knowledge of the potential role of RVs present in adolescents and adults patients with pneumonia is needed.

In general, compared to conventional viral diagnostic methods (culture, antigen detection, and serological assays), PCR-based methods are 2–5 times more sensitive to detect RVs [6]. Moreover, use of PCR has augmented detection of viruses that are difficult to identify with conventional methods, including human rhinovirus (HRV), human coronavirus (hCoV), human metapneumovirus (hMPV), and human bocavirus [7,8]. Recently, development of several multiplex assays has enabled simultaneous detection of up to 15 different viruses, and use of these tests is becoming standard for identification of respiratory viruses [9-11].

In an attempt to better characterize the viral etiology of CAP in adolescents and adults, a multi-center, prospective surveillance was conducted in 12 general hospitals of Beijing, from November 2010 to April 2012, using a multiplex RT-PCR assay covering all common RVs associated with CAP [12]. We also sought to evaluate age and severity of disease related to different pathogens.

## Methods

### Study setting and design

A prospective study was conducted in 12 general hospitals in Beijing, covering 80% of 30 million citizens, as described in former report [13]. Between November 2010 and April 2012, patients (aged 14 years or above) who came to the hospitals during daytime and met the inclusion criteria of CAP [3] were enrolled. Patients with HIV infection; neutropenia or chemotherapy; pregnant; known or suspected active tuberculosis, no informed consents or specimens were excluded. The study was approved by institutional review board in Beijing Chao-Yang Hospital (project approval number: 10-KE-49). Written informed consents were provided by all adults and the parents of patients aged less than 18 years.

### Patient characteristics and CAP scoring system

The following data were recorded on enrollment: age, gender, smoking status, comorbid illnesses and antimicrobial treatment prior to enrollment, duration of symptoms prior to visit, clinical symptoms, physical examination, chest X-ray or computed tomography (CT) scan pattern, blood analysis and antimicrobial and antiviral treatment. All surviving patients were followed-up by telephone after discharge for four weeks, symptoms and signs were recorded daily. Pneumonia severity index (PSI) score classes were assigned according to the authors’ original designations [14]. PSI classes were specified as follows: low risk = I-II, moderate risk = III, high risk = IV-V.

### Microbiological evaluation

Microbiological examination was performed in throat swab, sputum, urine and blood, at the central laboratory (Clinical Microbiological Laboratory of Beijing Chao-Yang Hospital). The etiology was considered definite if one of the following criteria was met: (1) positive urinary antigen for *Legionella pneumophila* (LP, Binax Now *L pneumophila* urinary antigen test; Trinity Biotech, Bray, Ireland); (2) positive urinary antigen for *Streptococcus pneumoniae* (Binax Now *S pneumoniae* urinary antigen test; Emergo Europe, The Netherlands); (2) positive bacterial culture from blood. The etiology was considered probable if one of the following criteria was met: (1) detection of RVs in throat swabs by RT-PCR using a Seeplex RV Detection Kit (Seegene Biotechnology Inc., Seoul, Korea) according to manufacturer’s instructions, including respiratory syncytial virus (RSV) types A and B, influenza virus (IFV) types A and B, parainfluenza virus (PIV) types 1, 2, 3 and 4, HRV, enterovirus (EV), hCoV types 229E, NL63, OC43 and HKU1, hMPV, and adenovirus (AdV), bocavirus; (2) purulent sputum (defined as an adequate quality sputum sample with > 25 leukocytes and < 10 epithelial cells per × 100 magnification field) with compatible findings of Gram staining; (3) detection of *Mycoplasma pneumoniae* (MP) in throat swabs by PCR as previously reported [15].

### Statistical analysis

Categorical variables were described with counts and percentages. Data for continuous variables were presented as mean (SD) or median (IQR) where appropriate. The proportions of individuals in each age and PSI groups diagnosed with each pathogen of interest were compared using χ^2^ tests (SPSS for Windows 13.0).

## Results

### Patient characteristics

**Figure 1:**
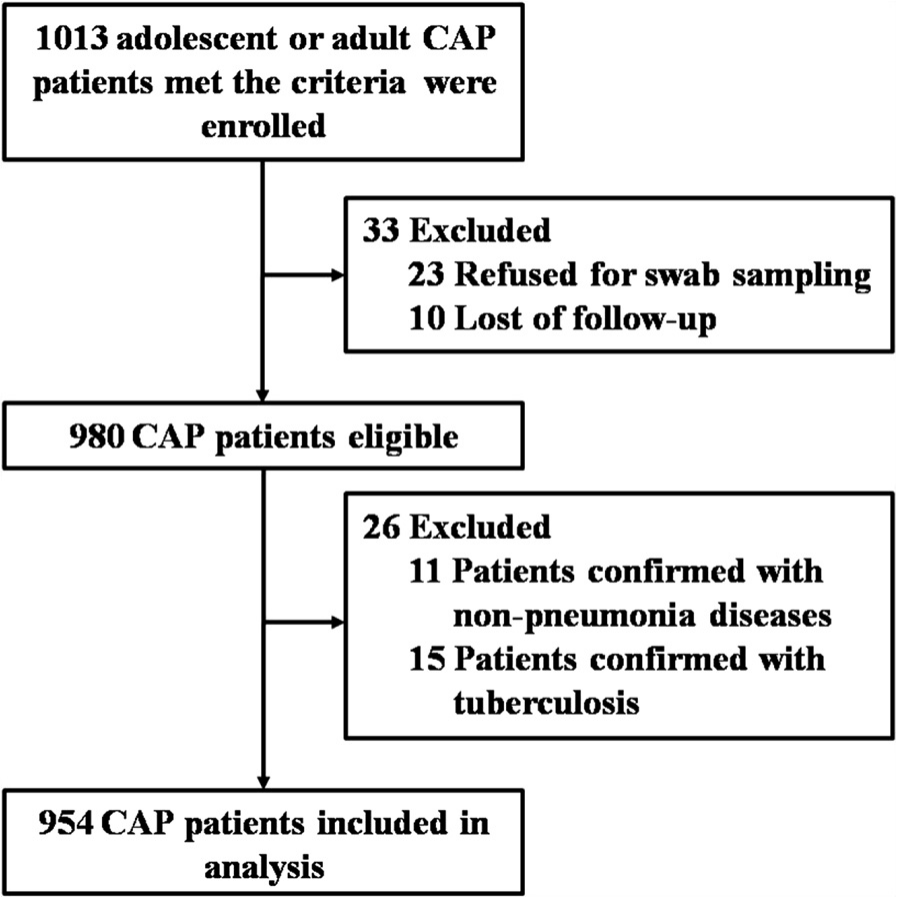
**Participants screening and enrollment.**

As seen in Figure 1, a total of 1013 adult CAP patients met the criteria were screened. Because of failing in sampling or follow-up, or confirmed with tuberculosis or non-pneumonia diseases (including lung cancer and etc.), 59 patients were excluded. Finally, etiological and clinical analysis was conducted on 954 patients (94.2%), and 56.6% were males. The mean (SD) age was 45.2 (19.5) years (range 14–94), 184 (19.4%) were aged ≥ 65 years.

The main demographic and clinical characteristics of the study population are summarized in Table 1. One hundred and eighty-four (19.3%) patients had comorbidities record, such as coronary heart diseases (8.1%), diabetes (7.6%), chronic obstructive pulmonary disease (2.3%) and etc. 254 (26.6%) patients had smoking history. Within one year, 40 (4.2%) and ten (1%) patients had received influenza and *streptococcus pneumonia* vaccine. Six hundred and twenty-seven (65.7%) patients were hospitalized, including six (0.6%) in ICUs. Six hundred and twenty-five patients (65.5%) had received antimicrobial treatment prior to enrollment. Common symptoms of the patients were fever (92%), cough (91.7%) and sputum production (70.4%). After admission, 918 patients (96.2%) and 26 patients (2.7%) received antimicrobial and antiviral treatment. Median (IQR) score of PSI was 42 (36). Four patients (0.4%) died within four weeks.

**Table 1:** **Epidemiological and clinical characteristics of study population**

**Characteristics**	**n (%)**
Total number of patients	954
Demographic data	
Age (years), mean ± SD	45.2 ± 19.5
Gender male	540 (56.6)
Comorbidities	184 (19.3)
Coronary heart diseases	77 (8.1)
Diabetes	73 (7.6)
Chronic obstructive pulmonary disease	22 (2.3)
Smoking (present or past)	254 (26.6)
Influenza vaccine received within 1 year	40 (4.2)
*Streptococcus pneumonia* vaccine received within 1 year	10 (1)
Site of care	
Outpatients	327 (34.3)
Inpatients	627 (65.7)
Ward	621 (65.1)
ICU	6 (0.6)
Antibiotics received before enrollment	625 (65.5)
Symptoms	
Fever	878 (92)
T_max_ (°C), mean ± SD	39.0 ± 0.7
Cough	875 (91.7)
Sputum	672 (70.4)
Shortness of breath	137 (14.4)
Chest pain	116 (12.2)
Laboratory findings	
Procalcitonin (n = 130), median (IQR)	0.17 (0.42)
C reactive protein (n = 671), median (IQR)	42.5 (86.8)
WBC count (×10^9^/L), median (IQR)	7.6 (4.7)
WBC > 10 (×10^9^/L)	257 (26.9)
WBC < 4 (×10^9^/L)	61 (6.4)
Antimicrobial treatment after enrollment	
Antibiotics	918 (96.2)
Antivirals	26 (2.7)
Length of hospital stay (days)	9 (6–14)
PSI, median (IQR)	42 (36)
Death	4 (0.4)

### Distribution of pathogens

Etiological diagnoses were established in 393 patients (41.2%), as shown in Table 2. The positive rate of RVs was 27.5%. IFVA was dominant (9.9%, 94/954). There were 60 cases of pH1N1, 30 cases of seasonal H3N2 (sH3N2) and four cases of both subtypes. Seven patients infected with pH1N1 had received influenza vaccine within one year. The detection rate of HRV was 4.3%, and followed by AdV, 4.2%; hMPV, 1.8%; PIV1, 1.7%; PIV3, 1.5%; PIV2, 1.2%; and IFVB, EV, RSVs, hCoV and PIV4 were rarely detected (<1%). No bocavirus was detected.

**Table 2:** **Etiology of study population with CAP**

**Pathogen identified**	**n (%)**
At least one pathogen	393 (41.2)
Respiratory viruses (RVs)	262 (27.5)
Influenza virus A	94 (9.9)
Pandemic H1N1 (pH1N1)	60 (6.3)
Seasonal H3N2 (sH3N2)	30 (3.1)
pH1N1 and sH3N2	4 (0.4)
Human rhinovirus	41 (4.3)
Adenovirus	40 (4.2)
Human metapneumovirus	17 (1.8)
Parainfluenza virus type 1	16 (1.7)
Parainfluenza virus type 3	14 (1.5)
Parainfluenza virus type 2	11 (1.2)
Influenza virus B	6 (0.6)
Enterovirus	5 (0.5)
Respiratory syncytial virus type A	5 (0.5)
Respiratory syncytial virus type B	4 (0.4)
Human coronavirus types OC43/HKU1	4 (0.4)
Human coronavirus types 229E/NL63	4 (0.4)
Parainfluenza virus type 4	1 (0.1)
Bocavirus	0 (0)
Bacteria	219 (23.0)
*Mycoplasma pneumoniae*	168 (17.6)
*Legionella pneumophila*	4 (0.4)
Typical bacteria	47

Data are expressed as n (%).

The positive rate of bacteria was 23%. Atypical bacterial pathogens, including *Mycoplasma pneumoniae* and *Legionella pneumophila*, were detected in 168 (17.6%) and four (0.4%) patients. *Strectococcus pneumoniae* was detected by urine antigen test in fifteen patients. In 533 CAP patients available with bacterial culture, typical bacterial pathogens were detected in 32 patients, mainly consisted of *Klebsiella pneumoniae*, *Pseubomonas aeruginosa*, *Streptococcus pneumoniae* and *Haemophilus influenzae* (data not shown).

There were 257 patients with hyperleucocytosis, as shown in Table 1. Among these patients, 42 bacterial pathogens (including *Mycoplasma pneumomniae*, *Streptococcus pneumoniae*, *Haemophilus influenzae* and etc.) and 62 viral pathogens (including human rhinovirus, influenza virus A, adenovirus and etc.) were detected. Mean (SD) score of PSI of hyperleucocytosis group was significantly higher than that of non-hyperleucocytosis group (53.75 ± 25.03 v.s. 43.98 ± 25.37, p < 0.001).

Two or more causative agents were found in 75 patients (7.9%), as shown in Table 3. The common associations among dual infections were: a RV and a bacterium in 48 cases (64%), two RVs in 11 cases (14.7%) and two bacteria in six cases (8%). Triple, quadruple and quintuple infections were detected. IFV A, detected in 26 cases, was the most frequently RV in co-infections. PIVs were co-detected in 23 cases, HRVs in thirteen, AdVs in eight, hCoVs in five, RSVs and hMPVs in four, IFVs B in three, and EV in one.

**Table 3:** **Distribution of co-infections**

**Associations**	**n (%)**
**Dual infections**	**65 (6.8)**
**RV + Bacterium**	**48**
IFV A + Bacterium	**19**
HRV + Bacterium	9
PIVs + Bacterium	8
AdV + MP	4
hCoVs + MP	2
IFV B + MP	2
RSVs + MP	2
hMPV + Bacterium	2
**RV + RV**	**11**
IFV A + hCoVs	2
IFV A + PIVs	2
HRV + PIVs	2
hMPV + PIVs	2
AdV + RSV A	1
AdV + PIVs	1
AdV + hCoVs	1
**Bacterium + Bacterium**	**6**
**Triple infections**	**8 (0.84)**
IFV A (sH3N2) + PIV1 + PIV2	1
IFV A (sH3N2) + PIV2 + PIV3	1
IFV A (pH1N1) + two Bacteria	1
HRV + PIV1 + PIV3	1
HRV + two bacteria	1
PIV1 + EV + bacterium	1
PIV1 + PIV3 + bacterium	1
IFV B + AdV + PIV3	1
**Quadruple infection**	**1 (0.1)**
PIV1 + PIV3 + two bacteria	1
**Quintuple infections**	**1 (0.1)**
HRV + PIV1 + PIV2 + PIV3 + RSV B	1
**Total**	**75/954 (7.9)**

Note: influenza virus (IFV) types A and B, human rhinovirus (HRV), adenovirus (AdV), human metapneumovirus (hMPV), parainfluenza virus (PIV) types 1, 2, 3 and 4, enterovirus (EV), respiratory syncytial virus (RSV) types A and B, human coronaviruses (hCoVs), *Mycoplasma pneumoniae* (MP).

Regarding on antiviral treatment, there were 16 patients received oseltamivir among 94 IFV A infections. The clinical analysis of antiviral treatment revealed that the median length of hospitalization of antiviral group was significantly shorter than that of non-antiviral group (5.5 days v.s. 8 days, p = 0.04).

Two of the four dead patients were positive with pH1N1, the other two were positive with *Streptococcus pneumoniae* and *Klebsiella pneumoniae*, and *Legionella pneumophila* and human rhinovirus.

### Microbial etiology in age groups

The study population was divided into four groups by age, 14 ~ 17 years (adolescents), 18 ~ 44 years (young adults), 45 ~ 64 years (old adults) and ≥ 65 years (elderly) as shown in Table 4, to look for pathogens more commonly associated with an age group. The microbial etiology was determined in 70%, 44.5%, 37.9% and 30.3% for four groups respectively (p < 0.001), and co-infection rate decreased (14%, 9%, 5.9% and 6.3% respectively). IFV A was more frequently found in old adults and elderly groups (p < 0.001). HRV was equally detected in all age groups. The frequency of AdV decreased according to the four groups (p < 0.001), and similar trend was found in PIV 2 (p = 0.053) and MP (p < 0.001). No hMPV was detected in adolescents, and no RSV or hCoV was found in adolescents and elderly adults. The frequencies of typical bacterial pathogens increased according to age group (p = 0.005).

**Table 4:** **Etiology of CAP according to age**

**Etiology**	**14 ~ 17 yrs (n = 50)**	**18 ~ 44 yrs (n = 443)**	**45 ~ 64 yrs (n = 272)**	**65 ~ 94 yrs (n = 189)**	**p value**
At least one pathogen	35 (70)	197 (44.5)	103 (37.9)	58 (30.3)	<0.001
Respiratory viruses (RV)	15 (30)	104 (23.5)	72 (26.5)	45 (23.8)	0.651
IFV A^d^	2 (4)	32 (7.2)	38 (14)	22 (11.6)	<0.001
pH1N1	2 (4)	20 (4.5)	24 (8.8)	14 (7.4)	0.743
sH3N2	0	9 (2)	14 (5.1)	7 (3.7)	
pH1N1 and sH3N2	0	3 (0.7)	0	1 (0.5)	
HRV	2 (4)	19 (4.3)	13 (4.8)	7 (3.7)	0.962
AdV^abcd^	7 (14)	23 (5.2)	5 (1.8)	5 (2.6)	<0.001
HMPV	0	6 (1.4)	6 (2.2)	5 (2.6)	0.524
PIV 1	2 (4)	8 (1.8)	2 (0.7)	4 (2.1)	0.236
PIV 3	2 (4)	7 (1.6)	3 (1.1)	2 (1.1)	0.384
PIV 2	1 (2)	9 (2)	1 (0.4)	0	0.053
IFV B	1 (2)	3 (0.7)	1 (0.4)	1 (0.5)	0.495
EV	1 (2)	3 (0.7)	1 (0.4)	0	0.301
RSVA	0	4 (0.9)	1 (0.4)	0	0.628
RSV B	0	4 (0.9)	0	0	0.351
HCoV OC43/HKU1	0	2 (0.5)	2 (0.7)	0	0.739
HCoV 229E/NL63	0	3 (0.7)	1 (0.4)	0	0.853
PIV 4	0	1 (0.2)	0	0	1.000
Bacteria	26 (52)	120 (27.1)	44 (16.2)	27 (14.3)	
MP^abcde^	25 (50)	106 (23.9)	24 (8.8)	13 (6.9)	<0.001
LP	1 (2)	2 (0.5)	1 (0.4)	0	0.262
Typical bacteria^cde^	0	12 (2.7)	19 (7)	14 (7.4)	0.005
Two or more pathogens	7 (14)	40 (9)	16 (5.9)	12 (6.3)	0.141

Data are expressed as n (%).

^a^: p < 0.05, 14 ~ 17 yrs group vs 18 ~ 44 yrs group;

^b^: p < 0.05, 14 ~ 17 yrs group vs 45 ~ 64 yrs group;

^c^: p < 0.05, 14 ~ 17 yrs group vs 65 ~ 94 yrs group;

^d^: p < 0.05, 18 ~ 44 yrs group vs 45 ~ 64 yrs group;

^e^: p < 0.05, 18 ~ 44 yrs group vs 65 ~ 94 yrs group;

Note: influenza virus (IFV) types A and B, human rhinovirus (HRV), adenovirus (AdV), human metapneumovirus (hMPV), parainfluenza virus (PIV) types 1, 2, 3 and 4, enterovirus (EV), respiratory syncytial virus (RSV) types A and B, human coronavirus (hCoV) types 229E, NL63, OC43 and HKU1, *Mycoplasma pneumoniae* (MP) and *Legionella pneumophila* (LP).

### Microbial etiology according to severity score

To explore the association between pathogens and the severity score, patients were also separated into three groups according to severity score (PSI), as indicated in Table 5. In three groups, though pathogens detection rates were different, RVs ranked first (25.1%, 19.5% and 30.4%, respectively). All RVs were detected in low risk group. The frequency of IFV A increased along with severity (from 9.4% and 8.8% to 17.9%). Significant difference (p = 0.018) was found in subtypes of IFV A among PSI groups, especially for pH1N1 (p < 0.05). Similar trend was found in the distribution of typical bacterial pathogens (p = 0.001). The distributions of HRV, AdV and hMPV in three groups were comparable. The frequency of MP decreased in moderate- and high-risk groups, whereas that of the mixed etiology decreased first (5.3%), then increased (8.9%).

**Table 5:** **Etiology of CAP according to PSI**

	**PSI I-II (n = 785)**	**PSI III (n = 113)**	**PSI IV-V (n = 56)**	**p value**
At least one pathogen	333 (42.5)	35 (29)	25 (44.6)	0.219
Respiratory viruses (RV)	197 (25.1)	22 (19.5)	17 (30.4)	0.267
IFV A	74 (9.4)	10 (8.8)	10 (17.9)	0.118
pH1N1^bc^	42 (5.4)	8 (7.1)	10 (17.9)	0.018
sH3N2	28 (3.6)	2 (1.8)	0	
pH1N1 and sH3N2	4 (0.5)	0	0	
HRV	33 (4.2)	5 (4.4)	3 (5.4)	0.854
AdV	36 (4.6)	3 (2.7)	1 (1.8)	0.542
HMPV	14 (1.8)	1 (0.9)	2 (3.6)	0.391
PIV 1	15 (1.9)	1 (0.9)	0	0.668
PIV 3	13 (1.7)	0	1 (1.8)	0.445
PIV 2	10 (1.3)	1 (0.9)	0	1.000
IFV B	6 (0.8)	0	0	1.000
EV	4 (0.5)	1 (0.9)	0	0.624
RSVA	4 (0.5)	1 (0.9)	0	0.624
RSV B	4 (0.5)	0	0	1.000
HCoV OC43/HKU1	4 (0.5)	0	0	1.000
HCoV 229E/NL63	4 (0.5)	0	0	1.000
PIV 4	1 (0.1)	0	0	1.000
Bacteria	187 (23.9)	17 (15.1)	13 (23.2)	
MP^a^	157 (20)	6 (5.3)	5 (8.9)	<0.001
LP	2 (0.3)	2 (1.8)	0	0.146
Typical bacteria^ab^	28 (3.6)	9 (8)	8 (14.3)	0.001
Two or more pathogens	64 (8.2)	6 (5.3)	5 (8.9)	0.562

Data are expressed as n (%).

^a^: p < 0.05, PSI I-II vs III;

^b^: p < 0.05, PSI I-II vs IV-V;

^c^: p < 0.05, PSI III vs IV-V;

Note: influenza virus (IFV) types A and B, human rhinovirus (HRV), adenovirus (AdV), human metapneumovirus (hMPV), parainfluenza virus (PIV) types 1, 2, 3 and 4, enterovirus (EV), respiratory syncytial virus (RSV) types A and B, human coronavirus (hCoV) types 229E, NL63, OC43 and HKU1, *Mycoplasma pneumoniae* (MP) and *Legionella pneumophila* (LP).

## Discussion

To our knowledge, this is the largest scale investigation of common RV infections in China in adolescents and adults with CAP, using PCR-based method. The CAP patients in our group were different from other CAP studies with specific feathers as below, (1) young, mean (SD) age was 45.24 (19.478) years; (2) 82.3% of the patients’ PSI classes wereI-III; (3) low numbers of ICU admission and deaths; (4) 9 of 12 teaching hospitals functioned as primary care facilities. Our results indicated that 27.5% of CAP patients have evidence of viral infection. It has been generally assumed that for respiratory infections due to viruses, the optimal specimen is the nasopharyngeal aspirate, rather than throat swabs we used here [16]. Actually, the detection rate was higher than 22% reported by Ruuskanen O et al. [6] based on 2910 CAP patients from 10 studies, also higher than 5–20% reported by other studies that had not used PCR-based assays [17].

IFV A was the first ranking viral pathogen. Among 94 cases, 68 (72.3%) had IFV A as the only identified pathogen, 20 (21.3%) had co-infection with bacteria, and six (6.4%) had co-infection with other RVs. Similar with other reports [18,19], the analysis of subtypes of IFV A revealed that pH1N1 virus was circulating along with sH3N2 virus, as this surveillance was carried out from the post-pandemic period of pH1N1 [20]. It has been reported that younger age and more severe respiratory compromise are key features of patients with pH1N1-associated pneumonia compared with seasonal influenza pneumonia [21]. Here, oppositely, pH1N1 infected patients were mostly distributed in patients older than 45 years, which was coincident with the findings reported by Viasus D et al. [22]. This upward shift in age distribution is probably due to a higher seropositivity against A (H1N1) pdm09 virus in young adult patients. On the other side, although IFV A infected cases distributed through all PSI groups, all cases in high risk group were determined as pH1N1 (accounting for 16.7%, p < 0.05). And 93.3% of sH3N2 positive patients were found in low risk group, without high-risk cases. Two of four dead patients were caused by pH1N1. These findings suggest that the severity of pH1N1-associated CAP is still higher [22]. Four co-infection cases of pH1N1 and H3N2 were presented with PSI I-II, the reason was unclear. Further analysis of corresponding viral load and antiviral treatment might be needed.

In agreement with our results (HRV, 4.3%), two recently reports using PCR assays suggested that HRV was important cause in CAP, with infection rates of 4.9% [23] and 7% [24]. Among 41 infections, 27 patients (65.9%) had HRV as the only identified pathogen, ten (24.4%) had co-infection with bacteria, and four (9.7%) had co-infection with other RVs. These results stand in contrast to those of previous studies, which reported that HRV commonly occurred with bacterial co-infection (approximately 41.9–57.1%) [25,26]. Moreover, it is reported that HRV single or mixed HRV /pneumococcal infection should be an independent cause of severe pneumonia [25-27]. In our study, oppositely, HRV infections occurred in all age and severity groups.

The incidence of adenovirus was 4.2%, which was in the upper scale of the range of < 1% - 4% as reported [12]. Although most infected cases are self-limited, adenovirus is recognized as one of the first viruses clearly linked with pneumonia [12]. David Lieberman’s team had found that 1.6% of adults CAP patients caused by adenovirus, and no such cases had been detected in healthy controls or non-pneumonia low respiratory tract infection cohort [23]. Few studies reported on the co-infection of adenovirus with other pathogens, here, we found four with MP and four with other RVs, accounting for 20%. Lauderdalea et al. reported that all infections of AdV were found in 17–44 years-old patients [28]. Similarly, the incidence was significantly higher in adolescents and younger adults (p < 0.001).

Regarding on other important viruses, the frequencies of PIVs, RSV, hMPV and HCoVs were lower than that of Dr. Andrew T. Pavia’s report [12]. Since most respiratory viruses are highly seasonal, the frequencies might be influenced by the variation in intensity of the study period, age of the population and region.

In this surveillance, CAP patients due to MP, an important atypical bacterial pathogen, were common in 14 ~ 44 years-old patients and recognized by PSI as a low-risk condition, which is in consistent with the previous findings [29,30]. Just as Roson et al. reported, the patients with bacterial infections were usually associated with increased severity and mortality [31]. The analysis of typical bacterial pathogens indicated that the incidence increased along with age and PSI classes of the patients. And one patient died from co-infection of *Streptococcus pneumoniae* and *Klebsiella pneumoniae*. However, bacterial pneumonia might be underdiagnosed in this study [1,32] due to the reasons: (1) we focused on viral pathogens, therefore patients unavailable with a blood or sputum culture were not excluded. Only 533 patients were detected for typical bacterial pathogens, though urinary antigen test was conducted in all patients; (2) the defined population was young and had low or moderate severity; (3) the rate of antimicrobial treatment before enrollment was high (65.5%).

Our study is subject to two limitations. First, as reported by David Lieberman [23], RVs could be detected in 7.1% of 450 controls without respiratory complaints. Healthy controls were not included to clarify the clinical significance of RVs, especially for the cases of triple and quadruple infections. Second, clinical relevance of viral load in the specimens could not be analyzed since the study was carried out in qualitative assays.

## Conclusions

The proportion of RV involvement in CAP is higher than previously reported. Influenza virus A pneumonia are usually found in patients older than 45 years, while, adenovirus pneumonia are commonly found in adolescents and young adult patients. Pandemic H1N1 virus is still recognized by PSI as a high-severity pathogen. The findings contribute baseline data on viral CAP study in China.

## Abbreviations

CAP: Community-acquired pneumonia
RV: Respiratory virus
PSI: Pneumonia severity index
SARS: Severe acute respiratory syndrome
IFV: Influenza virus
HRV: Human rhinovirus
AdV: Adenovirus
hMPV: Human metapneumovirus
PIV: Parainfluenza virus
EV: Enterovirus
RSV: Respiratory syncytial virus
hCoV: Human coronavirus
MP: *Mycoplasma pneumoniae*
